# Current status of breast cancer diagnosis and treatment capacity in county-level medical institutions in Sichuan Province, China: a cross-sectional study

**DOI:** 10.3389/fonc.2026.1847827

**Published:** 2026-05-20

**Authors:** Le Kang, Chi Du, Yulan Huang, Hao Chen, Xiang Tan

**Affiliations:** 1 Department of Hematology and Oncology, Anyue County People’s Hospital, Ziyang, Sichuan, China; 2 Department of Oncology and Hematology, Dujiangyan People’s Hospital, Chengdu, Sichuan, China; 3 Department of Colorectal, Gastrointestinal, and Burn Surgery, The Second People’s Hospital of Neijiang, Neijiang, Sichuan, China

**Keywords:** breast cancer, county-level medical institutions, diagnostic and therapeutic capacity, health equity, medication accessibility

## Abstract

**Objective:**

To evaluate the current capacity for breast cancer diagnosis and treatment in county-level medical institutions in Sichuan Province, China.

**Methods:**

This cross-sectional observational study was conducted between December 2023 and January 2024. A retrospective questionnaire was administered to 124 county-level medical institutions in Sichuan Province that reported providing breast cancer services. Survey domains included hospital grade and ownership, oncology accreditation, breast specialty section/group setup and staffing, breast cancer diagnostic and therapeutic techniques, AJCC TNM staging documentation rates, and access to antineoplastic and palliative care agents. Data were analyzed using descriptive statistics with SPSS version 22.0.

**Results:**

Of 124 institutions surveyed, six were excluded because they had not treated any breast cancer patients in 2023, leaving 118 for analysis. Tertiary hospitals accounted for 78.81% and public hospitals for 94.07%. 75.42% of institutions held qualifications for special outpatient services, while 57.63% possessed both recognition for special antineoplastic drugs and prescribing authority. An independent breast specialty section/group existed in 57.63% of institutions, but only 16.95% were equipped with a comprehensive multidisciplinary team. Simultaneous availability of breast ultrasound, CT, MRI, and mammography was reported by 33.90%; PET-CT and bone scintigraphy were available in 7.63% and 11.02%, respectively. Independent pathology departments existed in 71.19% of cases. Breast cancer surgery was performed in 86.44% of institutions and breast-conserving surgery in 44.07%; The radiotherapy provision rate was 38.98%. The AJCC TNM staging rate was 77.12%. Complete availability rates for conventional chemotherapy agents, anti-HER2 targeted therapies, endocrine agents, and CDK4/6 inhibitors were 33.90%, 22.88%, 47.46%, and 10.17%, respectively. Strong opioids for pain control and several key antiemetics showed variable accessibility.

**Conclusions:**

Breast cancer diagnostic and treatment capacity in county-level hospitals in Sichuan Province remains limited, reflecting common challenges in low- and middle-income countries (LMICs). Key deficiencies include underdeveloped dedicated breast oncology units, insufficient specialized personnel, restricted access to advanced imaging and innovative therapies (especially CDK4/6 inhibitors and anti-HER2 agents), and suboptimal availability of strong opioids and novel antiemetics for palliative care. Targeted policy interventions are needed to strengthen infrastructure, workforce, and drug accessibility at the county level. These findings provide valuable insights for improving breast cancer care in other resource-constrained settings globally.

## 1 Introduction

Breast cancer remains the most common malignancy among women globally and poses a significant public health challenge. According to GLOBOCAN 2022 estimates from the International Agency for Research on Cancer (IARC), approximately 2.3 million new cases and 670,000 deaths occurred worldwide in 2022, ranking first in incidence and a leading cause of cancer mortality among females (1). Marked regional disparities are evident: age-standardized incidence rates (ASIR) are substantially higher in high-Human Development Index (HDI) countries, such as those in North America and Western Europe, whereas age-standardized mortality rates (ASMR) remain disproportionately elevated in low- and middle-HDI regions, underscoring inequities in screening, early diagnosis, and treatment access (2).

In China, breast cancer is the most frequently diagnosed cancer in women, with an estimated 357,000 new cases and 75,000 deaths in 2022, accounting for 15.59% and 7.94% of all female cancer incidence and mortality, respectively (3). Although the overall incidence rate is lower than in many Western countries, a sustained upward trend has been observed, particularly in urban areas and among younger women (4). This rise is driven by rapid urbanization, adoption of Western lifestyles (including high-fat diets and reduced physical activity), and shifts in reproductive patterns (such as later marriage and childbirth) (4, 5). Notably, incidence in rural areas is also increasing, and approximately one-third of new cases are first diagnosed or managed at county-level medical institutions (county or county-level city hospitals). These facilities serve as the primary point of contact for the majority of rural breast cancer patients (6).

County-level institutions in China frequently face constraints on equipment, specialized human resources, and medication availability, which collectively hinder timely detection and standardized treatment (6). Consequently, the lack of robust empirical data from grassroots settings limits evidence-based policy formulation and timely responses to the growing burden of breast cancer in rural and county regions. Strengthening capacity at this level is critical for achieving the goals of the “Healthy China 2030” initiative and narrowing urban–rural health disparities (7). However, most existing research has concentrated on provincial or national cancer centers, leaving systematic evaluations of breast cancer diagnosis and treatment capacity at the county-level notably scarce (8).

This study employed a cross-sectional observational design to comprehensively assess breast cancer diagnosis and treatment capacity in 124 county-level medical institutions in Sichuan Province. The evaluation focused on institutional qualifications, specialty infrastructure and staffing, diagnostic and therapeutic techniques, AJCC TNM staging practices, and accessibility of antineoplastic and palliative care agents. By identifying key gaps and strengths, the study aims to provide granular, county-level evidence to inform targeted health policies and contribute to the development of more equitable breast cancer care systems in resource-constrained settings.

## 2 Methods

This was a multicenter, retrospective, cross-sectional observational study conducted in county-level medical institutions across Sichuan Province, China. These institutions primarily serve rural and semi-urban populations as secondary- and tertiary-tier medical centers within the regional hierarchical healthcare system.

The study employed a retrospective questionnaire-based survey design. A structured questionnaire was developed to collect institutional-level data on breast cancer diagnosis and treatment capacity. Participating institutions were asked to report service provision and resource availability based on their actual clinical practice and records from 2023, rather than hypothetical scenarios. The inclusion criteria for the institutions were as follows: (1) county-level medical institutions located in Sichuan Province; (2) institutions that claimed to provide breast cancer diagnosis and treatment services in their hospital registration information or annual reports; and (3) institutions willing to participate in the survey and provide relevant retrospective data for 2023. The exclusion criteria were: (1) institutions that had not treated any breast cancer patients in 2023; and (2) questionnaires with incomplete data or evident logical inconsistencies.

Between December 2023 and January 2024, the questionnaire was administered to 124 county-level medical institutions in Sichuan Province that had reported providing breast cancer services. To ensure the data were representative of active clinical service, institutions were excluded if they had not treated any breast cancer patients in 2023. Consequently, six institutions were excluded, resulting in a final cohort of 118 institutions for descriptive analysis.

Hospital administrators or designated oncology department representatives completed the survey on behalf of each institution. Each respondent provided information regarding one institution, including hospital grade and ownership, oncology accreditation status, establishment of breast specialty sections or groups, staffing levels, availability of diagnostic and therapeutic techniques, AJCC TNM staging documentation practices, and accessibility of antineoplastic agents and palliative care medications. All data reflected real-world institutional capacity and service delivery in 2023.

The survey collected detailed information on infrastructure (e.g., imaging modalities, pathology services, radiotherapy equipment), surgical capabilities (including breast-conserving surgery), multidisciplinary team configuration, and medication formularies (including conventional chemotherapy, anti-HER2 targeted therapies, endocrine agents, CDK4/6 inhibitors, and supportive/palliative drugs). All participating institutions provided informed consent for data sharing.

Data were retrospectively collected and analyzed using descriptive statistics. Categorical variables are presented as frequencies and percentages. No inferential statistical tests were performed, as the study aimed to describe current capacity rather than test hypotheses. Analyses were conducted using SPSS version 22.0.

This study was conducted in accordance with the Declaration of Helsinki. As the survey collected institutional-level, de-identified aggregate data without the direct involvement of human subjects or access to identifiable individual patient records, it was deemed exempt from formal ethical approval by the relevant institutional review boards. However, informed consent was obtained from all participating institutions (represented by hospital administrators or designated oncology department representatives) prior to their participation in the survey.

## 3 Results

**Figure 1:**
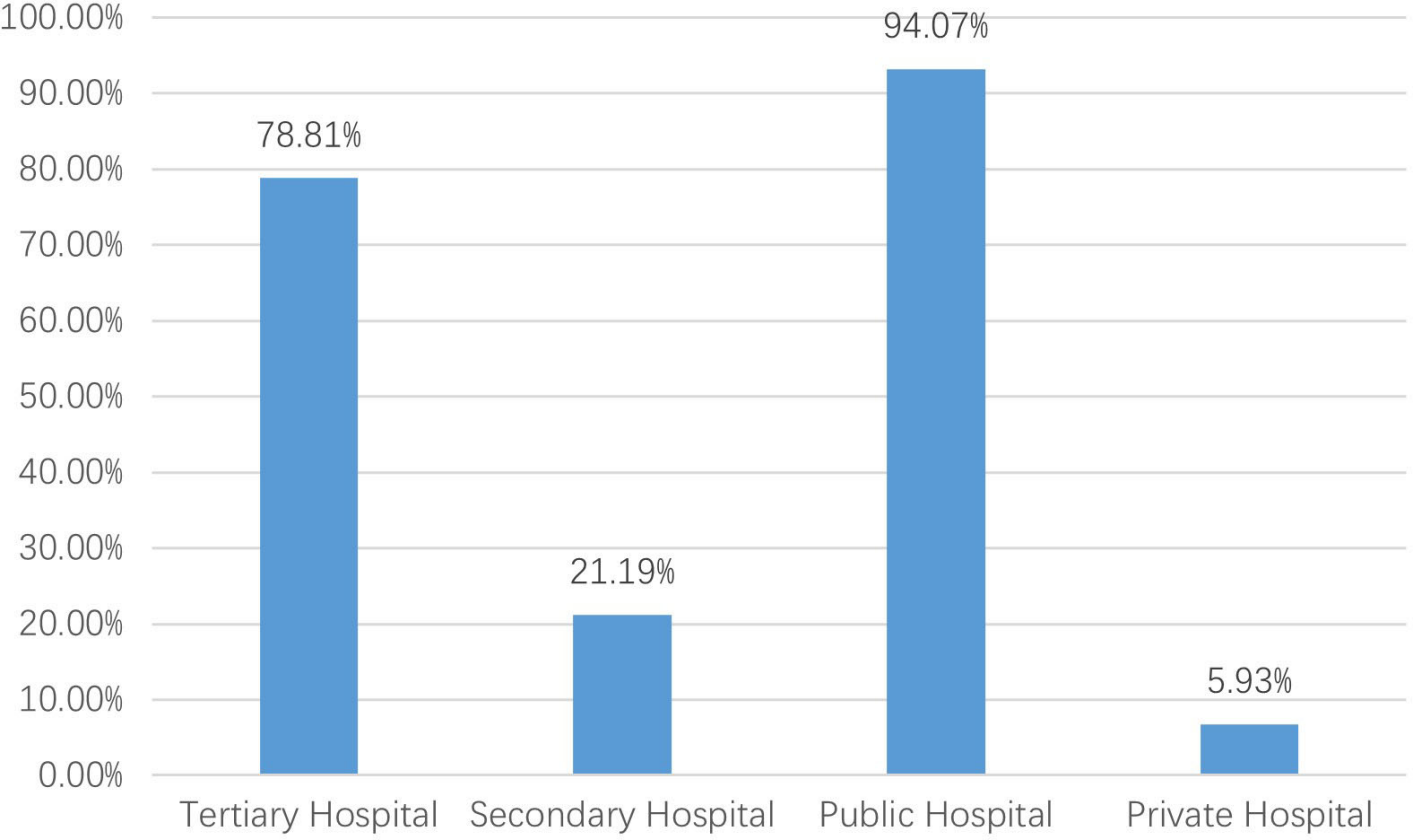
Hospital grade and ownership.

**Figure 2:**
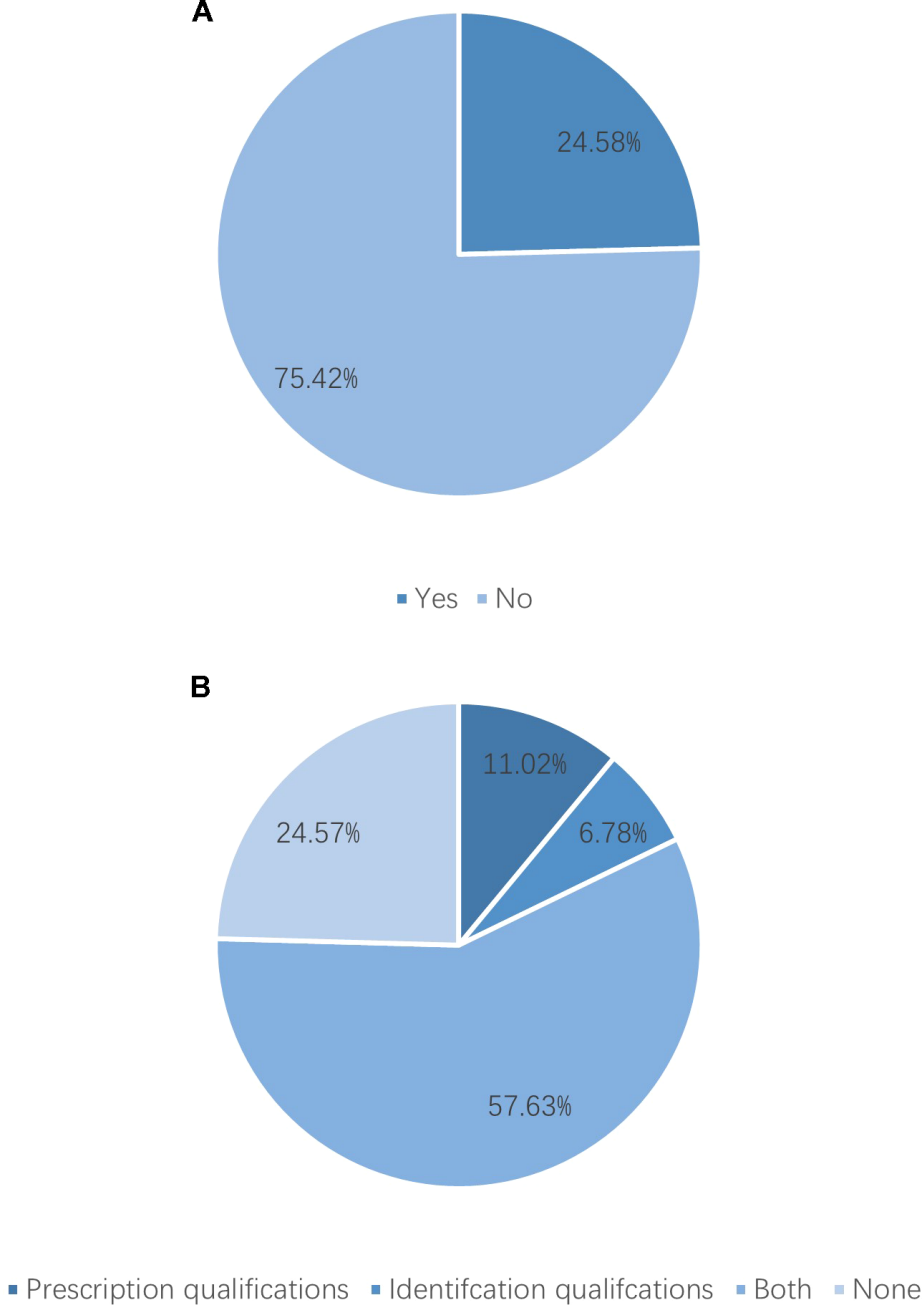
**(A)** Special outpatient qualifications. **(B)** Special antineoplastic drug identification and prescription qualifications.

**Figure 3:**
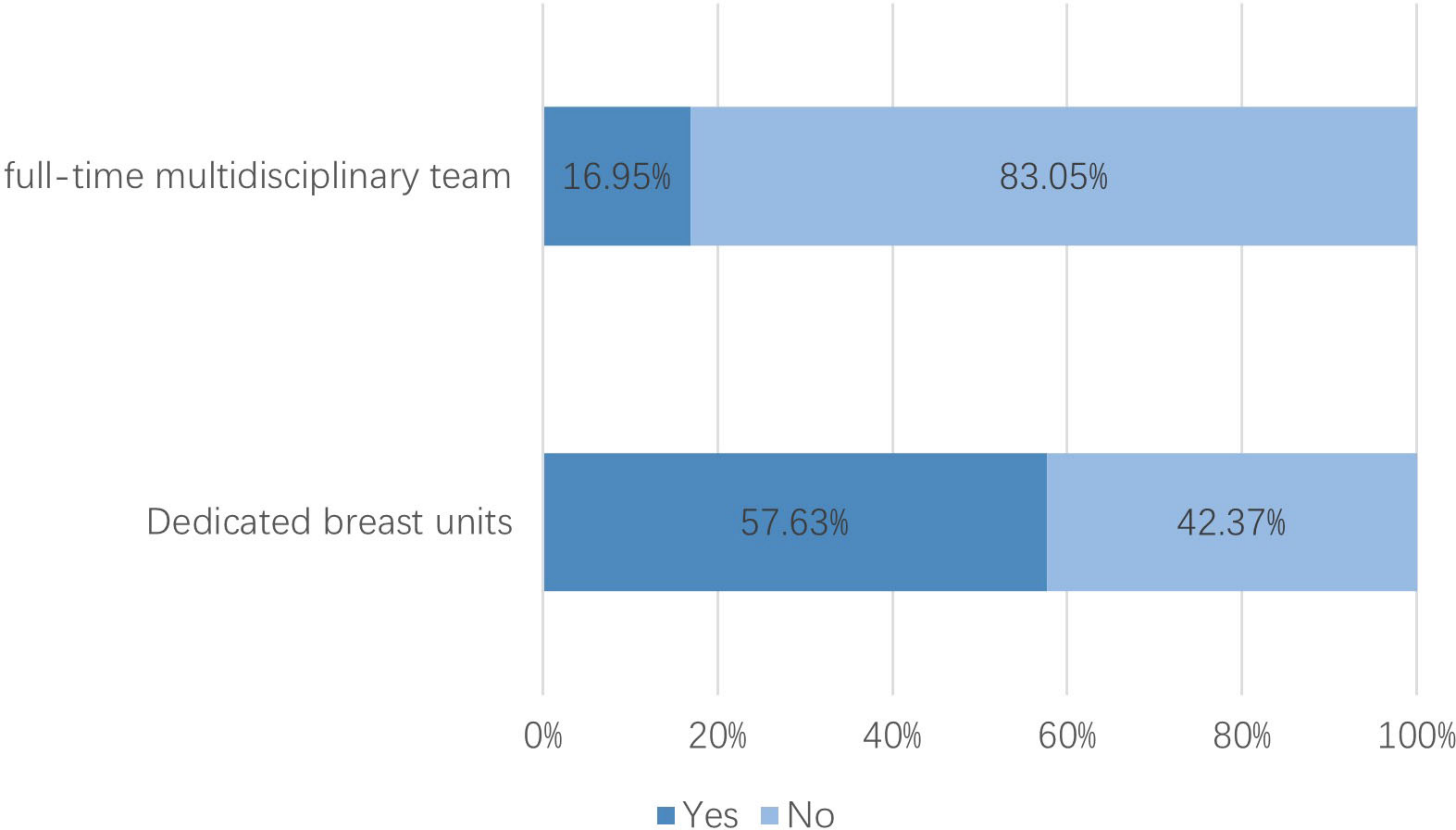
Availability of dedicated breast section/group and staffing.

**Figure 4:**
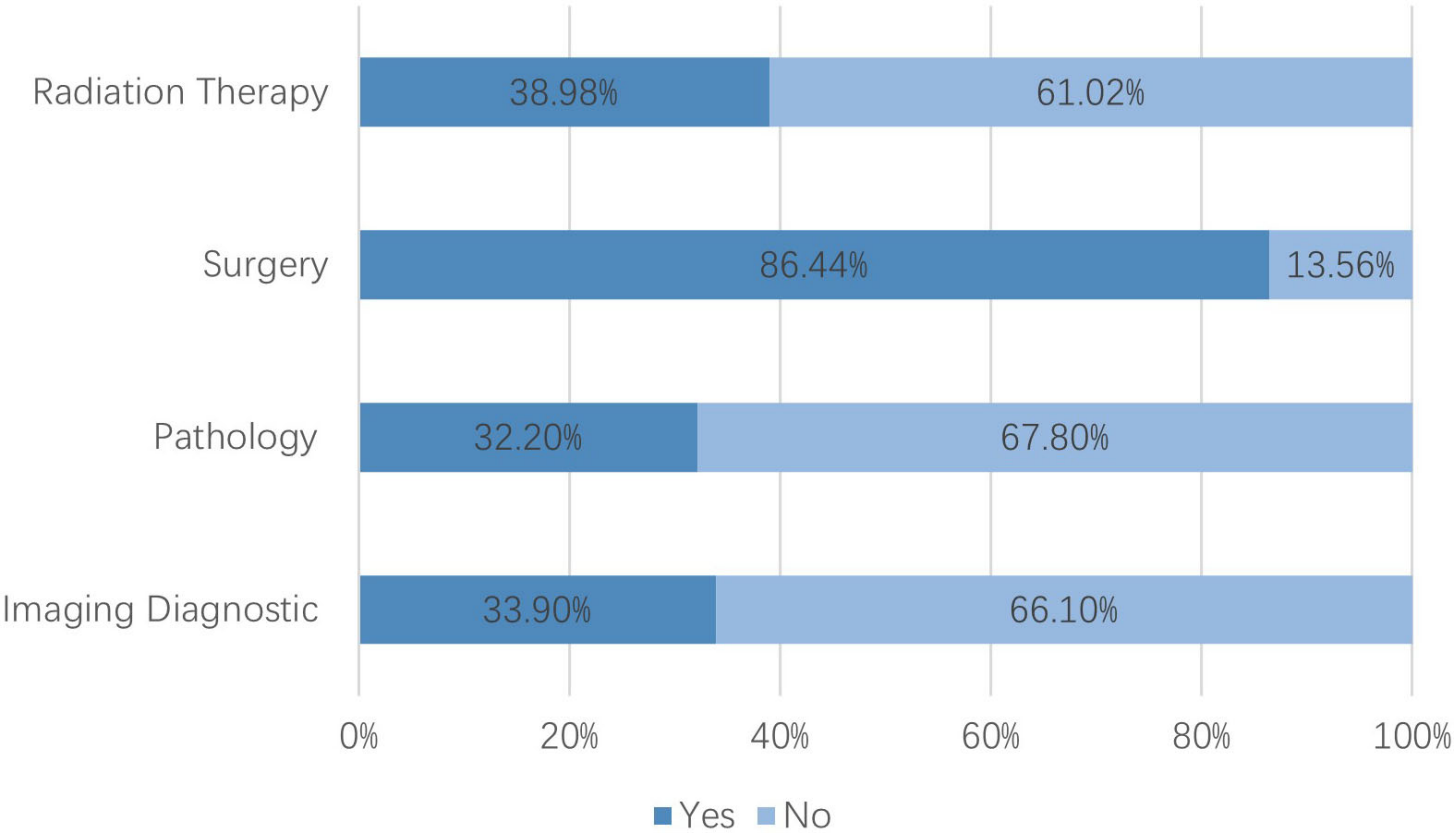
Breast cancer diagnostic and therapeutic capabilities.

**Figure 5:**
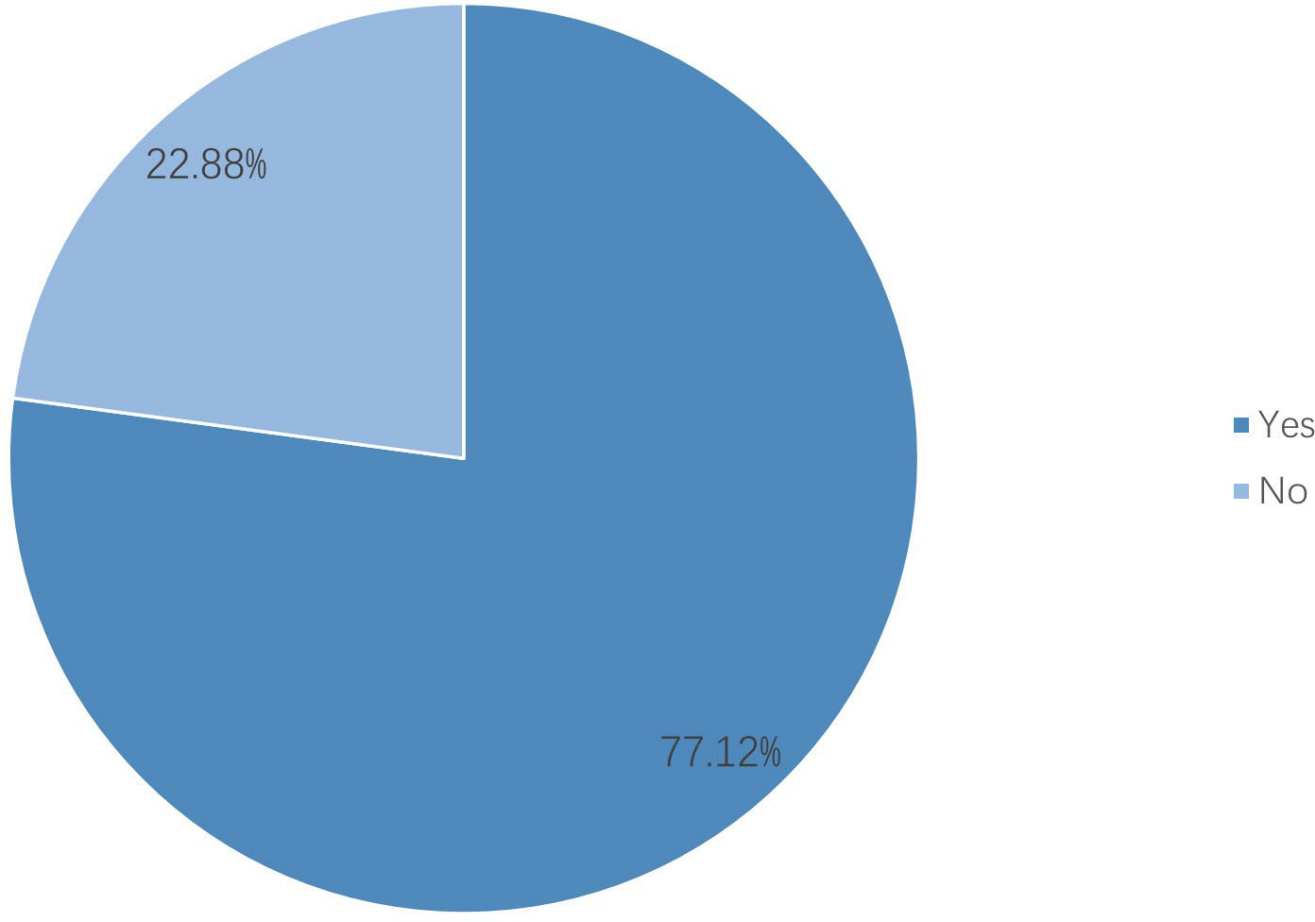
AJCC TNM staging rate.

**Figure 6:**
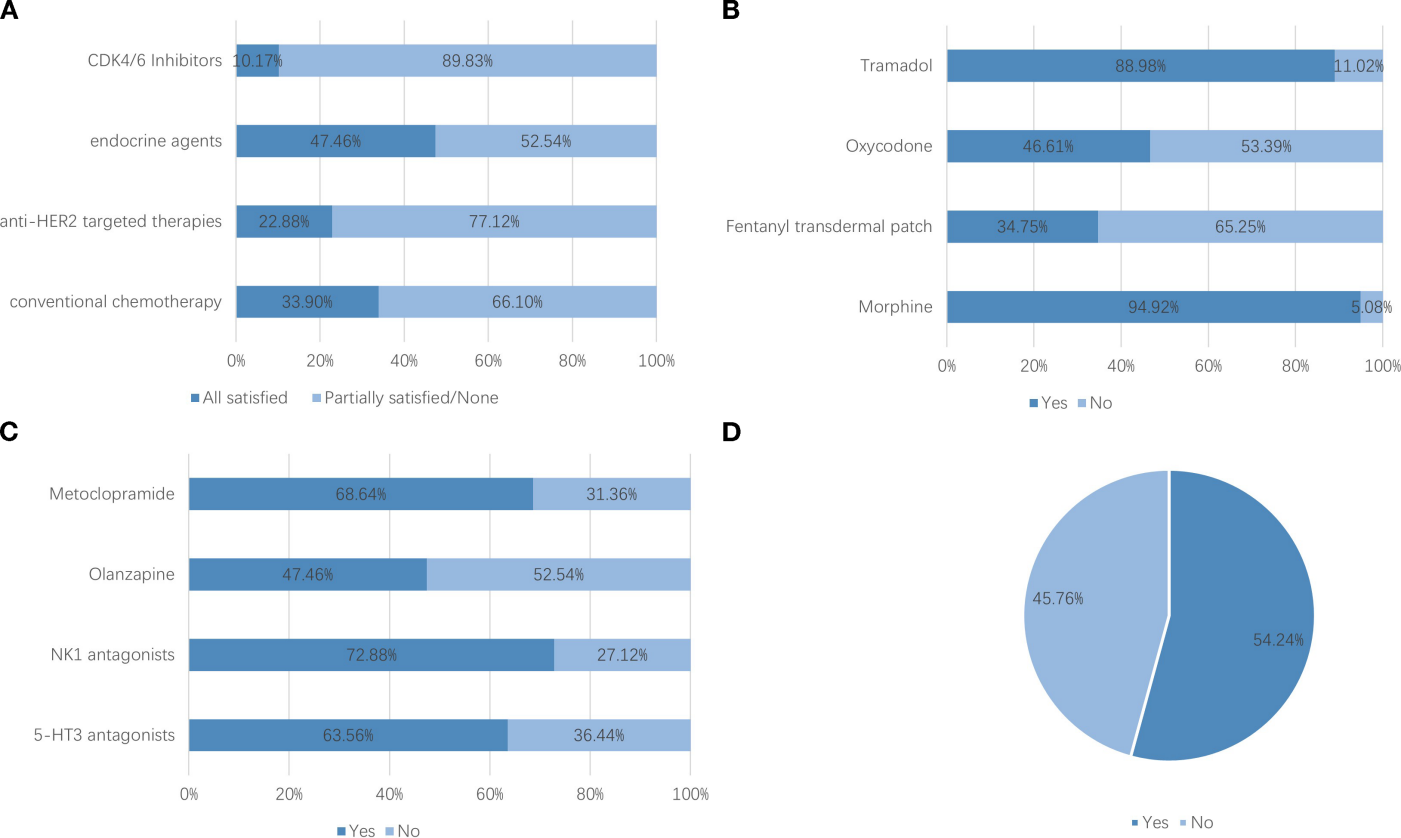
**(A)** Antineoplastic accessibility. Note: CDK4/6 inhibitors surveyed included abemaciclib, ribociclib, dalpiciclib, and palbociclib; endocrine agents included GnRH analogues, aromatase inhibitors, tamoxifen/toremifene, and fulvestrant; anti-HER2 agents included trastuzumab, pertuzumab, pyrotinib, T-DM1, and T-DXd; conventional chemotherapy included taxanes, anthracyclines, and platinum agents. **(B)** Analgesics accessibility. **(C)** Antiemetics accessibility. **(D)** Drugs for hematologic toxicity accessibility.

After excluding six institutions that had not treated breast cancer patients in 2023, 118 valid responses were analyzed. 1. Hospital grade and ownership: Tertiary hospitals comprised 78.81% and public hospitals 94.07% (**Figure 1**). 2. Oncology qualifications: 75.42% held Special outpatient qualifications; 57.63% possessed both Special antineoplastic drug identification and prescription qualifications, and 17.80% had only one of them. (**Figures 2A, B**, see **Supplementary Material**). 3. Breast specialty and staffing: While 57.63% of hospitals had established a breast specialty section/group, only 16.95% were equipped with a comprehensive multidisciplinary team comprising a breast cancer specialists, breast care nurses, oncology clinical pharmacists, radiotherapy technologists, and medical physicists (**Figure 3**; see **Supplementary Material**). 4. Diagnostic and therapeutic capabilities: Simultaneous performance of breast ultrasound, CT, MRI, and mammography was possible in 33.90%; PET-CT and bone scintigraphy availability were 7.63% and 11.02%, respectively. Independent pathology departments existed in 71.19%, but integrated biopsy, frozen section, paraffin histology, and immunohistochemistry services were available in only 32.20%. Breast cancer surgery was offered by 86.44% (breast-conserving surgery 44.07%) and radiotherapy by 38.98% (**Figure 4**; see **Supplementary Material**). 5. AJCC TNM staging rate: 77.12% (**Figure 5**). 6. Medication availability: Complete availability rates were 33.90% for conventional chemotherapy agents, 22.88% for anti-HER2 targeted therapies, 47.46% for endocrine agents, and 10.17% for CDK4/6 inhibitors (41.53% stocked only one CDK4/6 inhibitor) (**Figure 6A**; **Supplementary Material**). Analgesic availability was 88.98% for tramadol, 94.92% for morphine, 34.75% for fentanyl transdermal patches, and 46.61% for oxycodone (**Figure 6B**). Antiemetic availability was 63.56% for 5-HT3 antagonists, 72.88% for NK-1 antagonists, 47.46% for olanzapine, and 68.64% for metoclopramide (**Figure 6C**). Drugs for hematological toxicity were available in 54.24% of institutions (**Figure 6D**).

## 4 Discussion

This cross-sectional study systematically evaluated the capacity for breast cancer diagnosis and treatment in 124 county-level medical institutions across Sichuan Province, China. By assessing institutional characteristics, specialty infrastructure, diagnostic and therapeutic modalities, staging practices, and medication accessibility, the findings delineate both foundational strengths and critical gaps in primary-care breast cancer services. These results provide granular, county-level evidence that fills an important gap in the literature, which has predominantly focused on provincial or national cancer centers (8).

County-level institutions demonstrate clear strengths in infrastructure development and routine surgical practice. The distribution of hospital grades is reasonable, with 78.81% classified as tertiary facilities and 94.07% publicly owned, supporting equitable resource allocation and stability of care provision (9). Most hospitals hold oncology accreditation and qualifications for specialized outpatient services and antineoplastic prescribing. Breast cancer surgery is performed in 86.44% of institutions, breast-conserving surgery in 44.07%, and AJCC TNM staging is documented in 77.12% of cases. Basic imaging modalities (ultrasound, CT, and MRI) are widely available, providing essential support for initial and differential diagnosis and for determining appropriate therapeutic strategies (10, 11).

Nevertheless, substantial limitations remain in specialized team development, advanced diagnostic technologies, and drug accessibility. Although over half of institutions have established dedicated breast specialty sections or groups, only 16.95% maintain a comprehensive multidisciplinary team (MDT). This human-resource constraint impedes effective MDT implementation, a model consistently associated with improved outcomes in low- and middle-income countries (LMICs) (12, 13). Advanced diagnostic capacity is limited: simultaneous availability of breast ultrasound, CT, MRI, and mammography is present in only 33.90% of hospitals, while PET-CT and bone scintigraphy are available in only 7.63% and 11.02%, respectively. This limitation impedes the accurate evaluation of metastatic risk and the dynamic monitoring of treatment response—a situation consistent with healthcare resource constraints commonly observed in many LMICs (14–16). Independent pathology departments exist in 71.19% of institutions, yet complete workflows from biopsy to immunohistochemistry remain underdeveloped, limiting molecular subtyping and precision-medicine approaches (17, 18). Radiotherapy capacity is available in just 38.98% of facilities, restricting the broader application of breast-conserving surgery and standardized multimodal regimens (19).

Medication accessibility represents another critical barrier to optimal antineoplastic and palliative care. Complete availability of conventional chemotherapy agents reaches 33.90%, whereas anti-HER2 targeted therapies, endocrine agents, and CDK4/6 inhibitors are fully accessible in only 22.88%, 47.46%, and 10.17% of institutions, respectively. These shortfalls align with documented challenges in LMICs and rural China, where national medical insurance negotiations and centralized procurement policies have improved affordability, but local formulary management capacity, drug utilization assessment, reimbursement structures, and patients’ out-of-pocket affordability continue to constrain clinical use (20–24). Real-world evidence underscores the survival benefit of these agents; their limited availability at the county level may therefore directly compromise long-term outcomes (25–28). In palliative care, morphine and tramadol are relatively accessible, but transdermal fentanyl patches, oxycodone, and certain antiemetics or hematologic toxicity agents show lower availability, reflecting persistent global disparities in opioid and supportive-care access in resource-limited settings (29–33).

Contextual factors unique to Sichuan Province amplify these deficiencies. As a populous southwestern region with complex topography and marked urban–rural divides, the province relies heavily on county-level hospitals as the primary point of contact for rural patients (34, 35). Lower health literacy, reduced breast cancer awareness, and socioeconomic barriers contribute to delayed presentation and a higher proportion of advanced-stage disease (36–38). These realities help explain the apparent paradox of relatively high TNM staging rates coexisting with limited early-detection infrastructure (39, 40). Geographic isolation and economic constraints further hinder procurement and maintenance of high-cost equipment and medications, particularly radiotherapy and targeted therapies (41–44). Consequently, the standardized implementation of post-lumpectomy radiotherapy remains challenging within county-level healthcare systems.

From an international perspective, high-HDI countries have markedly reduced breast cancer mortality through systematic screening, standardized pathology, widespread radiotherapy access, and timely integration of precision therapies (12, 16, 19, 45). In contrast, LMICs—including some regions of China—continue to face shortages of trained personnel, inadequate medical equipment, limited access to essential medications, and non-standardized treatment practices (12, 13, 16, 18, 19, 29–31, 37, 41, 46, 47). By focusing explicitly on the county level, this study addresses a key evidence gap and supplies actionable data for region-specific policy formulation.

Targeted interventions are therefore urgently needed to strengthen county-level breast cancer care. Priorities should include central-government investment to expand access to radiotherapy and advanced imaging, systematic training programs to build breast oncology subspecialties and MDT models (12, 48), accelerated inclusion of innovative agents in the National Reimbursement Drug List (49, 50), and tailored rural screening and health-education initiatives supported by telemedicine and artificial intelligence-assisted diagnostics. Such policy-driven strategies will enhance the hierarchical healthcare system, reduce urban–rural disparities, and generate a replicable framework for other LMICs worldwide.

Finally, a limitation of this study is its reliance on an institutional-level cross-sectional survey design. Our focus was primarily on evaluating hospital infrastructure, hardware facilities, and medication accessibility. Consequently, we did not collect individual patient-level clinicopathological data, such as specific lines of therapy, prior treatments, or refractory/relapsed status. Future multicenter patient cohort studies are needed to further clarify the specific clinical characteristics and survival outcomes of these patients in county-level primary care settings.

## 5 Conclusions

The diagnostic and therapeutic capacity for breast cancer in county-level hospitals in Sichuan Province mirrors challenges commonly observed in LMICs worldwide. Principal limitations include the underdeveloped infrastructure of dedicated breast oncology units and insufficient allocation of specialized personnel; restricted access to advanced diagnostic modalities and innovative anticancer agents (particularly CDK4/6 inhibitors and anti-HER2 therapies), which impedes accurate molecular subtyping and personalized treatment approaches; and inadequate availability of strong opioids and novel antiemetics for palliative care. Improving breast cancer care at the county level will require policy-driven strategies to optimize resource allocation and build subspecialty capabilities—a framework with important implications for other resource-constrained settings globally.

## Data Availability

The original contributions presented in the study are included in the article/**Supplementary Material**. Further inquiries can be directed to the corresponding author.

## References

[1] Bray F Laversanne M Sung H Ferlay J Siegel RL Soerjomataram I . Global cancer statistics 2022: GLOBOCAN estimates of incidence and mortality worldwide for 36 cancers in 185 countries. CA: A Cancer J For Clin. (2024) 74:229–63. doi:10.3322/caac.21834. PMID: 38572751

[2] Kim J Harper A McCormack V Sung H Houssami N Morgan E . Global patterns and trends in breast cancer incidence and mortality across 185 countries. Nat Med. (2025) 31:1154–62. doi:10.1038/s41591-025-03502-3. PMID: 39994475

[3] Xu YY Zhang YH Ding LL Chen YS Wang J Yan YF . Epidemiological characteristics of female breast cancer in China and worldwide. Chin J Oncol. (2025) 47:1228–33. doi:10.3760/cma.j.cn112152-20241127-00538 41443734

[4] Tao X Li T Gandomkar Z Brennan PC Reed WM . Incidence, mortality, survival, and disease burden of breast cancer in China compared to other developed countries. Asia-Pacific J Clin Oncol. (2023) 19:645–54. doi:10.1111/ajco.13958. PMID: 37026375

[5] Guidelines for the diagnosis and treatment of advanced breast cancer in China (2024 edition). Cancer Innovation. (2025) 4(6):e70032. doi:10.1002/cai2.70032. PMID: 41281946 PMC12629868

[6] Guideline for the management pathway and quality control of breast cancer prevention and treatment in China's counties. Cancer Innovation. (2025) 4(3):e70005. doi:10.1002/cai2.70005. PMID: 40151334 PMC11939006

[7] Han B Zheng R Zeng H Wang S Sun K Chen R . Cancer incidence and mortality in China, 2022. J Natl Cancer Center. (2024) 4:47–53. doi:10.1016/j.jncc.2024.01.006. PMID: 39036382 PMC11256708

[8] Zeng H Chen W Zheng R Zhang S Ji JS Zou X . Changing cancer survival in China during 2003–15: a pooled analysis of 17 population-based cancer registries. Lancet Global Health. (2018) 6:e555–67. doi:10.1016/s2214-109x(18)30127-x. PMID: 29653628

[9] Goodair B Reeves A . The effect of health-care privatisation on the quality of care. Lancet Public Health. (2024) 9:e199–206. doi:10.1016/s2468-2667(24)00003-3. PMID: 38429019

[10] Amin MB Greene FL Edge SB Compton CC Gershenwald JE Brookland RK . The eighth edition AJCC cancer staging manual: Continuing to build a bridge from a population‐based to a more “personalized” approach to cancer staging. CA: A Cancer J For Clin. (2017) 67:93–9. doi:10.3322/caac.21388 28094848

[11] Yao S Xiong B Tuo JY Qin Y Meng FD Xia YF . Survival analysis of Malignant tumors in cancer registration areas of Hubei province in China, 2013 to 2015. Zhonghua Zhong Liu Za Zhi. (2023) 45:1051–6. doi:10.3760/cma.j.cn112152-20230403-00145 38110313

[12] Paluch-Shimon S Biganzoli L Torode J Mason G Kim SB Chidebe RCW . Ensuring access to multidisciplinary care in advanced breast cancer: a global expert review and call-to-action for 2025–2035 (Goal 4). Breast. (2025) 84:104609. doi:10.1016/j.breast.2025.104609. PMID: 41298012 PMC12683121

[13] Nietz S Edge J Buccimazza I Demetriou G Tunmer M Smilg J . Establishing requirements for breast centers in low- and middle-income countries: a South African perspective. JCO Global Oncol. (2025) 11:e2500168. doi:10.1200/go-25-00168. PMID: 40532139

[14] Zhang H Zheng J Hu N Liu Y Yang G Liu M . The status of nuclear medicine in China: the first official national survey. Eur J Nucl Med Mol Imaging. (2024) 51:2172–8. doi:10.1007/s00259-024-06687-w. PMID: 38561514

[15] Bastidas JF Martínez de Bourio-Allona M Roteta Unceta Barrenechea A Rodríguez-Fraile M Sancho L . PET/CT in breast cancer. Rev Esp Med Nucl Imagen Mol (Engl Ed). (2025) 44:500139. doi:10.1016/j.remnie.2025.500139. PMID: 40914389

[16] Hussain HK Rajeev R DeStigter KK Gulani V . Global cancer imaging access: Addressing barriers and harnessing innovations. Radiology: Imaging Cancer. (2025) 7(6):e250019. doi:10.1148/rycan.250019. PMID: 41134140 PMC12670022

[17] Long Z Qiu Y Long Z Jin Z . Epidemiology of breast cancer in Chinese women from 1990 to 2021: a systematic analysis and comparison with the global burden. BMC Cancer. (2025) 25(1):3. doi:10.1186/s12885-024-13336-w. PMID: 39757149 PMC11702233

[18] Tolentino-Rodriguez L Chkeir M Pofagi V Ahindu I Toniolo J Erazo A . Breast cancer characteristics in low- and middle-income countries: An umbrella review. Cancer Epidemiol. (2025) 96:102797. doi:10.1016/j.canep.2025.102797. PMID: 40081022

[19] Abdel-Wahab M Giammarile F Carrara M Paez D Hricak H Ayati N . Radiotherapy and theranostics: a lancet oncology commission. Lancet Oncol. (2024) 25:e545–80. doi:10.1016/s1470-2045(24)00407-8. PMID: 39362232

[20] Li W Lu W Chen H Zhang C Wang M Zheng F . Access to innovative anticancer medicines in China: a national survey on availability, price and affordability. BMJ Open. (2024) 14(4):e077089. doi:10.1136/bmjopen-2023-077089. PMID: 38670605 PMC11057311

[21] Ma R Liu Y Ding B Sun Y Yao H Yu M . A survey of availability, price, and affordability of innovative negotiated anticancer medicines: a retrospective study in Jiangsu Province, eastern China. BMC Public Health. (2025) 25(1):2313. doi:10.1186/s12889-025-23570-x. PMID: 40610972 PMC12224675

[22] Yao Y Zhu Y Jing Z Gao L Zhu H Wang J . Availability, price and affordability of anticancer medicines in Jiangsu Province, China: a cross-sectional survey study. Front Public Health. (2025) 13:1729325. doi:10.3389/fpubh.2025.1729325. PMID: 41487632 PMC12756471

[23] Zhao D Zhou Z . Intended and unintended impacts of ‘4+7’ volume-based drug procurement on the use of drugs in China: a natural experimental study. Healthcare. (2025) 13(6):686. doi:10.3390/healthcare13060686. PMID: 40150536 PMC11941950

[24] Hu Q Kuai L Xu D Lyu L . Impact of six rounds of national drug price negotiation on availability, cost, use, and prices of targeted drugs in China. BMC Health Serv Res. (2025) 25(1):1421. doi:10.1186/s12913-025-13575-y. PMID: 41162975 PMC12574164

[25] Khalid H Murtaza M Jaber Amin M . Cyclin-dependent kinase 4/6 inhibitor in hormone receptor-positive, human epidermal growth factor receptor 2-negative breast cancer: global challenges and Tibremciclib perspective. JCO Glob Oncol. (2025) 11:e2500457. doi:10.1200/go-25-00457. PMID: 41343746

[26] Harbeck N Brufsky A Rose CG Korytowsky B Chen C Tantakoun K . Real-world effectiveness of CDK4/6i in first-line treatment of HR+/HER2- advanced/metastatic breast cancer: updated systematic review. Front Oncol. (2025) 15:1530391. doi:10.3389/fonc.2025.1530391. PMID: 40129925 PMC11931418

[27] Antonini M Mattar A Pereira da Costa Pinheiro DJ Maia IB Teixeira MD Amorim A . Disparities in access to anti-HER2 therapies in neoadjuvant chemotherapy: a prognostic analysis based on real-world data comparing Brazil's public and private healthcare systems. Breast. (2025) 80:104417. doi:10.1016/j.breast.2025.104417. PMID: 39983435 PMC11893340

[28] Lapuchesky L Bortz M Waisberg F Enrico D Bruno L Ostinelli C . CDK4/6 inhibitors outcomes in patients with advanced breast cancer based on HER2-low expression. J Clin Oncol. (2022) 40:1056. doi:10.1200/jco.2022.40.16_suppl.1056

[29] Andres EB Yo V Balasubramanian I Poco L Ozdemir S Manalo MF . Opioid access among advanced cancer patients in low- and middle-income countries in Asia. J Pain Symptom Manage. (2024) 68:352–9. doi:10.1016/j.jpainsymman.2024.06.020. PMID: 38964427

[30] Knaul FM Farmer PE Krakauer EL De Lima F Bhadelia A Jiang Kwete X . Alleviating the access abyss in palliative care and pain relief—an imperative of universal health coverage: the Lancet Commission report. Lancet. (2018) 391:1391–454. doi:10.1016/s0140-6736(17)32513-8. PMID: 29032993

[31] Vallath N Rajagopal MR Perera S Khan F Paudel BD Tisocki K . Access to pain relief and essential opioids in the WHO South-East Asia Region: challenges in implementing drug reforms. WHO South East Asia J Public Health. (2018) 7:67–72. doi:10.4103/2224-3151.239416. PMID: 30136663

[32] Knaul FM Arreola-Ornelas H Kwete XJ Bhadelia A Berterame S Connor SR . Distributed opioids in morphine equivalent: a global measure of availability for palliative care. J Pain Symptom Manage. (2025) 69:204–15. doi:10.1016/j.jpainsymman.2024.10.026. PMID: 39490604 PMC11778810

[33] Erfani P Okediji RL Mulema V Cliff ERS Asante-Shongwe K Bychkovsky BL . Advancing global pharmacoequity in oncology. JAMA Oncol. (2025) 11(1):55–59. doi:10.1001/jamaoncol.2024.5032. PMID: 39541207

[34] Fort AL Ding S Zhou Y . County medical community, medical insurance package payment, and hierarchical diagnosis and treatment—empirical analysis of the impact of the pilot project of compact county medical communities in Sichuan Province. PloS One. (2024) 19(4):e0297340. doi:10.1371/journal.pone.0297340 38578741 PMC10997099

[35] Liu L Liu C Duan Z Pan J Yang M . Factors associated with the inter-facility transfer of inpatients in Sichuan province, China. BMC Health Serv Res. (2019) 19(1):329. doi:10.1186/s12913-019-4153-7. PMID: 31122226 PMC6533730

[36] Ji Y Zhou F Qu H Wang J Liu M Liang W . 1570P factors and impacts of delayed presentation for county-level patients with breast cancer in a real-life setting in China. Ann Oncol. (2024) 35:S951. doi:10.1016/j.annonc.2024.08.1632. PMID: 38826717

[37] Hewage SA Samaraweera S Joseph N Kularatna S Gunawardena N . Presentation, diagnosis and treatment delays in breast cancer care and their associations in Sri Lanka, a low-resourced country. Clin Oncol (R Coll Radiol). (2022) 34:598–607. doi:10.1016/j.clon.2022.05.007. PMID: 35672184

[38] Zhu L Zhou Q Huang Z Yang Y Yang Y Du Y . Factors influencing breast cancer awareness in rural southwest China: a cross-sectional study. Int J Women's Health. (2024) 16:509–18. doi:10.2147/ijwh.s453857. PMID: 38533523 PMC10964776

[39] Wang H Hua X Yao N Zhang N Wang J Anderson R . The urban-rural disparities and associated factors of health care utilization among cancer patients in China. Front Public Health. (2022) 10:842837. doi:10.3389/fpubh.2022.842837. PMID: 35309211 PMC8931518

[40] Sepassi A Li M Zell A Chan A Saunders IM Mukamel DB . Rural-urban disparities in colorectal cancer screening, diagnosis, treatment, and survivorship care: a systematic review and meta-analysis. Oncologist. (2024) 29:e431–46. doi:10.1093/oncolo/oyad347. PMID: 38243853 PMC10994268

[41] Gnangnon FHR Lawani I Knight S Parenté A Dossou FM Totah T . Assessing the continuum of care in Sub-Saharan African hospitals performing surgery for breast cancer: a secondary analysis of the GlobalSurg 3 study. BMC Cancer. (2024) 24(1):1529. doi:10.1186/s12885-024-13267-6. PMID: 39695461 PMC11656810

[42] Yang F Yuan C Lu S Xiao H Gao L . Assessment of radiotherapy and diagnosis resources allocation in Shanghai, China. Radiat Med Prot. (2024) 5:207–12. doi:10.1016/j.radmp.2024.05.006. PMID: 38826717

[43] Bhadouriya R Rohit RV Selvakumar JP Yadav P Patel TA Jain B . Disparities in refusal of adjuvant radiation for breast-conserving therapy in the USA. Ann Surg Oncol. (2025) 32:4034–40. doi:10.1245/s10434-025-17152-9. PMID: 40119190

[44] Laskar SG Sinha S Krishnatry R Grau C Mehta M Agarwal JP . Access to radiation therapy: From local to global and equality to equity. JCO Global Oncol. (2022) 8:e2100358. doi:10.1200/go.21.00358. PMID: 35960905 PMC9470145

[45] Chou CY Shen TT Wang WC Wu MP . Favorable breast cancer mortality-to-incidence ratios of countries with good human development index rankings and high health expenditures. Taiwanese J Obstetrics Gynecology. (2024) 63:527–31. doi:10.1016/j.tjog.2023.11.012. PMID: 39004480

[46] Subedi R Houssami N Nickson C Nepal A Campbell D David M . Factors influencing the time to diagnosis and treatment of breast cancer among women in low- and middle-income countries: a systematic review. Breast. (2024) 75:103714. doi:10.1016/j.breast.2024.103714. PMID: 38522173 PMC10973645

[47] Afaya A Ramazanu S Bolarinwa OA Yakong VN Afaya RA Aboagye RG . Health system barriers influencing timely breast cancer diagnosis and treatment among women in low and middle-income Asian countries: evidence from a mixed-methods systematic review. BMC Health Serv Res. (2022) 22(1):1601. doi:10.1186/s12913-022-08927-x. PMID: 36587198 PMC9805268

[48] Wang N Li L Han L Jiang S Xiong Y Wang F . Application of multidisciplinary diagnosis and treatment (MDT) clinical teaching model in the clinical teaching of some undergraduates of breast diseases in surgery. BMC Med Educ. (2025) 25(1):982. doi:10.1186/s12909-025-07540-w. PMID: 40597159 PMC12217188

[49] Bucher HC Chammartin F . Strengthening health technology assessment for cancer treatments in Europe by integrating causal inference and target trial emulation. Lancet Regional Health - Europe. (2025) 52:101294. doi:10.1016/j.lanepe.2025.101294. PMID: 40255411 PMC12008668

[50] Glaus CEG Serra-Burriel M Dusetzina SB Vokinger KN . Time from approval to reimbursement of new drugs: a comparative analysis between the United States, England, Germany, France, and Switzerland (2011-2022). Ann Intern Med. (2024) 177:1442–4. doi:10.7326/annals-24-00614. PMID: 39222511

